# Population dynamics of an invasive bird parasite, *Philornis downsi* (Diptera: Muscidae), in the Galapagos Islands

**DOI:** 10.1371/journal.pone.0224125

**Published:** 2019-10-18

**Authors:** Charlotte E. Causton, Roger D. Moon, Arno Cimadom, Rebecca A. Boulton, Daniel Cedeño, María Piedad Lincango, Sabine Tebbich, Angel Ulloa

**Affiliations:** 1 Charles Darwin Research Station, Charles Darwin Foundation, Puerto Ayora, Santa Cruz Island, Galapagos Islands, Ecuador; 2 Department of Entomology, University of Minnesota, St. Paul, MN, United States of America; 3 Department of Behavioural Biology, University of Vienna, Vienna, Austria; 4 College of Life and Environmental Sciences, University of Exeter, Cornwall, United Kingdom; 5 Facultad De Ciencias Agrícolas, Universidad Central Del Ecuador, Quito, Pichincha, Ecuador; Universita degli Studi di Milano-Bicocca, ITALY

## Abstract

The invasive parasitic fly, *Philornis downsi* (Muscidae), is one of the greatest threats to the avifauna of the Galapagos Islands. The larvae of this fly feed on the blood and tissues of developing nestlings of at least 18 endemic and native birds. The aim of the current study was to investigate biotic and abiotic factors that may influence the population dynamics of this invasive parasite. To study the influence of vegetation zone and related climatic factors on fly numbers, a bi-weekly monitoring program using papaya-baited traps was carried out at a dry, lowland site and at a humid, highland site on Santa Cruz Island between 2012–2014. Female flies, a large proportion of which were inseminated and gravid, were collected throughout the year at both sites, indicating females were active during and between the bird breeding seasons. This is the first evidence that female flies are able to persist even when hosts are scarce. On the other hand, catch rates of male flies declined between bird breeding seasons. Overall, catch rates of *P*. *downsi* were higher in the drier, lowland habitat, which may be a consequence of host or resource availability. Time was a stronger predictor of adult fly numbers than climate, further suggesting that *P*. *downsi* does not appear to be limited by its environment, but rather by host availability. Seasonal catch rates suggested that populations in both habitats were continuous and multivoltine. Numbers of adult female flies appeared to be regulated chiefly by simple direct density dependence, and may be governed by availability of bird nests with nestlings. Nevertheless, confounding factors such as the existence of reservoir hosts that perpetuate fly populations and changes in behavior of *P*. *downsi* may increase the vulnerability of bird hosts that are already IUCN red-listed or in decline.

## Introduction

Avian parasite invasions have been responsible for many of the observed declines or extinctions in avian biodiversity worldwide [1,2]. Endemic avian species on oceanic archipelagos that are already threatened by habitat reduction and introduced predators are especially at risk from new parasite incursions [1,3,4]. Such is the case in the Galapagos Islands where the recent invasion of an avian nest parasitic fly, *Philornis downsi* (Diptera: Muscidae), has seriously affected the stability of indigenous Galapagos passerine populations [5,6,7]. This parasitic fly has a broad host range [8], which has enabled it to quickly establish new host-parasite relationships in the Galapagos Islands with at least 17 passerine species and one cuculiform species reported as new hosts [5].

Correlative and experimental studies on the impact of *P*. *downsi* clearly demonstrate that this invasive fly reduces the reproductive success of Galapagos landbirds and is the cause of substantial bird population declines over the last three decades [5,6]. The development of effective techniques to minimize the impacts of *P*. *downsi* is a high priority [9], but is impeded by significant gaps in our understanding of the fly’s ecology, in particular the environmental and biological factors that have played a role in its successful establishment in these novel ecosystems [5,9]. The aim of the current study was to close gaps in knowledge about the biotic and abiotic factors that influence the population dynamics of this invasive parasite.

*Philornis downsi* is native to mainland South America and was likely introduced to the Galapagos Islands from mainland Ecuador [10]. The earliest record in the archipelago dates back to 1964, but larvae were not reported in nests until 1997 [5,11]. Now, up to 100% of nests of 18 species of Galapagos landbirds are parasitized by this invasive fly [5,6,7]. The life cycle of *P*. *downsi* is closely tied to the development of its hosts. The non-parasitic adult flies lay their eggs in nests with bird eggs or nestlings [12,13]. Once chicks hatch, the young fly larvae typically feed inside the chicks’ nares or auditory canals for the first few days of the life cycle, after which they move to the base of the nest emerging at night to feed on the blood and tissues of nestlings [12]. Larvae pupate in the base of the nest and life cycle completion takes approximately 15–24 days from egg to adult [12,14,15].

*Philornis downsi* has been recorded on 15 of 17 of the largest islands of the Galapagos archipelago [5]. The large islands of Santa Cruz, San Cristobal, Floreana, and Isabela support continuous populations of *P*. *downsi*, but smaller islands (< 1 km^2^) appear to be unable to sustain *P*. *downsi* populations year round [5]. Factors that influence population numbers and the spatial distribution of adult *P*. *downsi* on the Galapagos Islands are not well understood. A particularly important question is what happens to *P*. *downsi* in the cool season when the majority of host species are not breeding [9].

In 2012 we established a multiple year monitoring program to address the following questions: (1) Do seasonal patterns in adult fly numbers, relative frequencies of males and females, and reproductive activity differ in lowland and highland regions on Santa Cruz Island? (2) Is variation in numbers related to weather, bird nesting activity, or other features of the two regions? (3) Are population growth and decline in both regions regulated by density dependence and weather effects or both?

## Materials and methods

### Study sites

Studies were carried out from June 2012 to December 2014 on Santa Cruz Island, Galapagos, Ecuador. One study site was in the island’s lowland region, at El Barranco (0°44’34.1”S, 90°18’10.4”W, elevation 15–41 m), where vegetation was predominantly *Opuntia* and *Jasminocerus* cacti, *Cordia lutea*, *Acacia* sp, and *Parkinsonia aculeata* trees. The second site was in the highland region at Los Gemelos (0° 37’82.0”S, 90°23’44.4”W, elevation 589–616 m), vegetated primarily by endemic *Scalesia pedunculata* forest. Much of the understory of this forest was covered in invasive blackberry, *Rubus niveus* Thunb. [16]. The lowlands and highlands on Santa Cruz are distinct vegetation zones and rainfall is typically much lower in the lowlands [17].

### Weather data

Daily weather measurements near the lowland site were provided by the Charles Darwin Research Station (CDRS) and recorded at Academy Bay, Puerto Ayora, Santa Cruz (0°44'37.06"S, 90°18'7.94"W, elevation: 2 m). Measurements near the highland study site were provided by Rolf Sievers and recorded at El Carmen, Santa Cruz (0°39´57.49"S, 90°22´35.04´´ W), a station at an elevation of ~ 410 m, 180–200 m lower than the highland study site.

### Fly traps

Trapping at both locations began at the end of the bird breeding season in June, 2012, and continued through two full breeding and non-breeding seasons in 2013 and 2014. Fly abundance was monitored at each site with McPhail traps (Naturquim, Ecuador). Each trap was baited with 200 ml of a blended papaya-based mixture of 600 g ripe papaya fruit, 75 g sugar and 4 l water, a mixture that is known to be attractive to *P*. *downsi* [18]. Traps were hung in trees 3–4 m above ground along transects at the selected study sites. The spatial distribution of the transects was based on the accessibility and homogeneity of the study area. Thirty traps were hung along a single transect at the lowland site on 6 June, 2012 (mean minimum distance between traps 21 ± 1m SE). Another 30 traps were set along two parallel transects (15 traps per transect) at the highland site on 29 June, 2012 (mean minimum distance between traps 19.8 ± 0.7m SE) with a minimum distance of 83m between transects. Traps were set out every 15 days at each study site with a one-day difference between sites. The last trapping dates were 25 December, 2014 at the lowland site and 25 December, 2014 at the highland site. At both sites, baited traps were left in place for seven days, but trapped flies were removed at day four and again at day seven to reduce fly decomposition. All flies were stored in 70% alcohol and later examined under a stereomicroscope (Zeiss Stereo Microscope 32 x) to determine species and sex.

### Reproductive status of female flies

Up to 20 females were haphazardly selected from each bi-weekly collection at the lowland site in 2013 and at both lowland and highland sites in 2014 to assess reproductive development and fecundity in the two populations. Under a stereomicroscope, each female was dissected to categorize reproductive development. A female was classified as “undeveloped” if she had only undifferentiated primary follicles, or differentiated follicles that were in early to late stages of oogenesis. A female was “gravid” if she had one or more fully developed eggs with dark, melanized chorions, and egg numbers were counted to quantify fecundity. Finally, spermathecae were squashed on a microscope slide to view white liquid sperm. A preliminary study had shown that spermathecae of females that had not been exposed to males were empty.

### Monitoring for *P*. *downsi* parasitism in nests

Between January through April in each of 2012, 2014 and 2015, a total of 184 Warbler Finch (*Certhidea olivacea*) nests and 158 Small Tree-finch (*Camarhynchus parvulus*) nests were monitored in an area of approximately 20 ha within the *Scalesia* forest at the highland site. Nest status was checked with a small endoscopic video camera (dnt Findoo 3.6) to determine date of failure (if chicks died) or fledging, following the methodology in Cimadom *et al*. [19]. After activity ceased, individual monitored nests were collected into plastic bags and dissected in the laboratory at CDRS to count immatures of *P*. *downsi* (larvae, pupae and empty puparia). In 2012, larvae and pupae were grouped separately in 90 mm diam. petri dishes for incubation at 27°C and 65% relative humidity until eclosion or death. In 2014 and 2015, larvae were provided with chicken blood, and pupae were isolated individually in muslin covered plastic cups (4.5 cm diam, at top, height 4.5 cm), following a protocol for rearing larvae in captivity [15]. Emerged flies were identified to sex and counted to quantify numbers of males and females.

### Ethics statement

The study was conducted in the protected areas of the Galapagos National Park. Permission to conduct this study was granted by the Directorate of the Galapagos National Park (Projects: PC-02-14, PC-04-15, PC-02-14, PC-10-15). Our study, including the nest monitoring of Darwin's finches, was purely descriptive, strictly non-invasive and based exclusively on behavioral observations. The nest monitoring is classified as non-animal experiments in accordance with the Austrian Animal Experiments Act (§ 2. Federal Law Gazette No. 501/1989).

### Data analysis

We analyzed the bi-weekly counts of male and female flies from 2012–2014 to test hypotheses that seasonal patterns in abundance of the two sexes differed through the two and a half years at the lowland and highland study sites, and that abundance was directly related to concurrent temperatures and precipitation levels. Similarly, we analyzed the data from dissected females trapped in 2013 and 2014 for evidence that reproductive activity was seasonal. Counts of male and female flies reared from nests were examined to test the hypotheses that relative frequencies of males and females were equal and seasonally invariant, and to determine if densities in nests during 2014 were related to concurrent trap catch rates at the highland site in that year. Finally, we applied time series methods to analyze changes in mean female fly abundance between consecutive generations to test hypotheses that population growth rates from one generation of females to the next (1) were related to population density, (2) differed between bird breeding and nonbreeding seasons, and (3) were related to weather effects.

### Seasonal abundance of adult flies in lowland and highland habitats

Bi-weekly counts of flies from all traps (*n* = 8,040 possible counts of males and females over all weeks) were converted to daily catch rates (DCRs) by dividing each by number of trapping days, which in most cases was 7 days. To formally assess whether catch rates of males *vs* females differed across the highland and lowland sites, and whether catch rates depended on local weather conditions, we fit statistical models using catch rates as the dependent variable, fixed effects of fly sex and site, the interaction between the two, and a random effect of trap location. To assess possible short-term effects of weather on catch rates, we calculated mean temperature (T,°C), mean humidity (RH, %), and mean daily rain (R, mm), averaged over days in each trapping interval and study site, and included each mean as a continuous covariate in the fly sex-site model. We tested all two-way interactions effects between all climatic variables and site and sex. Variance inflation factors (VIFs) calculated for weather variables were low (in [0–1]), suggesting that multicollinearity was not a problem. The *weights* function was used to compensate for heterogeneity of variances. Appropriate variance function structures for each independent variable were determined using Akaike’s Information Criterion (AIC) to determine model fit. The model was fit with the function *lme* in the package lme4 in R [20].

Daily catch rates were also analyzed with a general additive mixed model (GAMM) using R’s mgcv package to examine the influence of broader scale seasonal changes on the catch rate. The outcome variable was DCR, trap location was fitted as a random effect, and heterogeneity of variances was allowed for using the *weights* function. A Duchon spline was determined to be the best fit to the temporal variable (‘study weeks’). Spline type was chosen based on the model fit determined using AIC. We used GAMM in mgcv to determine the importance of broad scale seasonal and fine scale climatic changes on the catch rate. We ran several GAMM’s to determine how much of the temporal variation in catch rates could be accounted for by substituting fixed continuous effects of weather variables for ‘study weeks’. To do this we ran one model with only the smoothing term for ‘weeks’ as a predictor. We ran additional models that included only weather (fitted as linear or as a cubic spline) or both weather and weeks. The AIC was then used to determine if the weather or weeks model had stronger support, or if climate and time both contributed significantly to *P*. *downsi* catch rates, indicating that broad scale seasonal and finer scale temporal fluctuations both play a role in regulating *Philornis* catch rates. We ran additional GAMMs to test whether the same seasonal (weeks) and climatic (weather) processes influenced male and female trap rates in the same manner. To do this, separate splines were fitted for males and for females according to ‘weeks’ and ‘weather’. We repeated this process partitioning the splines by site, in order to test whether seasonal and climatic fluctuations in catch rate were consistent across the highland and lowland sites.

### Reproductive status of females

Counts of undeveloped and gravid females were analyzed with a binomial generalized linear mixed model (GLMM) to assess seasonal patterns in reproductive status, with fixed effects of site-years, months within site-years, and interactions. We detected overdispersion (dispersion parameter = 7.58), which we accounted for using an observation level random effect (OLRE [21]). Similarly, counts of eggs per gravid female were analyzed with a Poisson GLMM to compare egg loads in unmated and mated females, and among sampling months within site-years, with an ORLE (dispersion parameter = 7.33). Finally, proportions that were mated were analyzed using a binomial GLMM with site-years, months, reproductive stage, and matching mean daily log_2_ catch rate of females as predictors and an OLRE (dispersion parameter = 37.97). Mean daily log_2_ catch rate was included to see if mating frequencies were low when adult fly density was low (Allee effect). Candidate models were fit with the *glmer* procedure in R [20].

### Sex ratio of flies emerging from nests

We compared frequencies of female vs. male *P*. *downsi* emerging from Warbler Finch and Small Tree-finch nests among 2012, 2014 and 2015, and among weeks within years, by fitting generalized linear mixed models with an observation level random effect (dispersion parameter = 14.34; [21]). We also considered covariates of number of flies per nest, flies per chick, and fraction of flies sexed. The significance of individual model terms was examined with simultaneous Type II tests using the *Anova* procedure.

### Levels of nest parasitism and matching catch rates

Trapping of *P*. *downsi* coincided with nest monitoring at the highland site in 2014. To see if catch rates could predict numbers of larvae per nest, we selected mean log_2_ DCR from the week that most closely matched each nest’s egg hatching date, and then we fit a series of Poisson regression models, initially with categorical effects of hatching week and bird species, and covariates of chicks per nest, age of nest at fledging time or failure, matching log_2_ DCR, and possible pair-wise interactions. Candidate GLMMs were fit with *glmer* in R [20] using an OLRE to compensate for overdispersion (dispersion parameter = 15.77).

### Population dynamics of *P*. *downsi*

Difference equations were used to examine patterns in population growth and decline from one generation to the next. Initial analysis of abundance indicated generations overlapped, so we estimated calendar limits between consecutive generations with temperature-dependent phenology models for generation time (S1 Appendix). We then time-averaged catch rates between successive calendar limits to estimate average DCR for each generation, and then analyzed changes in those averages from one generation to the next using difference equations. We disregarded males, presuming that female fertility was not limited by lack of mating.

Two time series analysis methods were used to probe for evidence in the monitoring data that the introduced *P*. *downsi* population in the Galapagos Islands was regulated and stationary [22,23]. First, we examined autocorrelation functions (ACFs) for mean catch rates among consecutive generations. An ACF for an unregulated population would damp slowly toward zero over progressively longer series of generations (lags), and perhaps become negative, whereas the ACF from a regulated population would damp faster and remain near zero or perhaps oscillate around zero. Second, we examined partial rate correlation functions (PRCF) [24]. Values for lags of 1 or more generations reveal the “dimensionality” of candidate regulatory mechanisms, whatever they may be. A significantly negative value at lag 1 would indicate dynamics were 1-dimensional, as with direct density dependence of growth rate on maternal density, whereas significant values with lags of 2 or greater would indicate delays longer than one generation, perhaps involving interspecific competition or predator-prey relations. We calculated ACFs and PRCFs for the series of generational mean trap catch rates at each study location with Nonlinear Time Series Modeling (NLTSM) described in Turchin [23].

Results indicated that density dependent, 1-generation, first order processes were chiefly responsible for governing population dynamics (however we note that autocorrelation coefficients for the 4 generation lag did at times reach statistical significance, suggesting that more subtle interspecific interactions also play a role here (see below). To examine the density dependent (lag 1) dynamics further we used analysis of covariance to assess a full model for relationships between per-capita population growth rate and fixed effects of parental density, study site, concurrent generational mean temperature and rainfall, and whether growth was during the bird breeding season or not. Growth rate was defined as a change in mean log density from one generation (mothers) to the next (daughters). In theory, if a population is regulated, per-capita growth rate will be positive when density is below carrying capacity, and negative if above. Carrying capacity was estimated from the x-intercept of the regression of growth on density [22]. Generational mean temperatures and rainfall levels were calculated by averaging each weather variable across days in each generation and at each study site. Terms in the full model were deleted, based on lack of AIC support, and then fitted regression equations for minimally sufficient models were used to estimate equilibrium densities at the two study sites.

## Results

### Weather data

**Fig 1:**
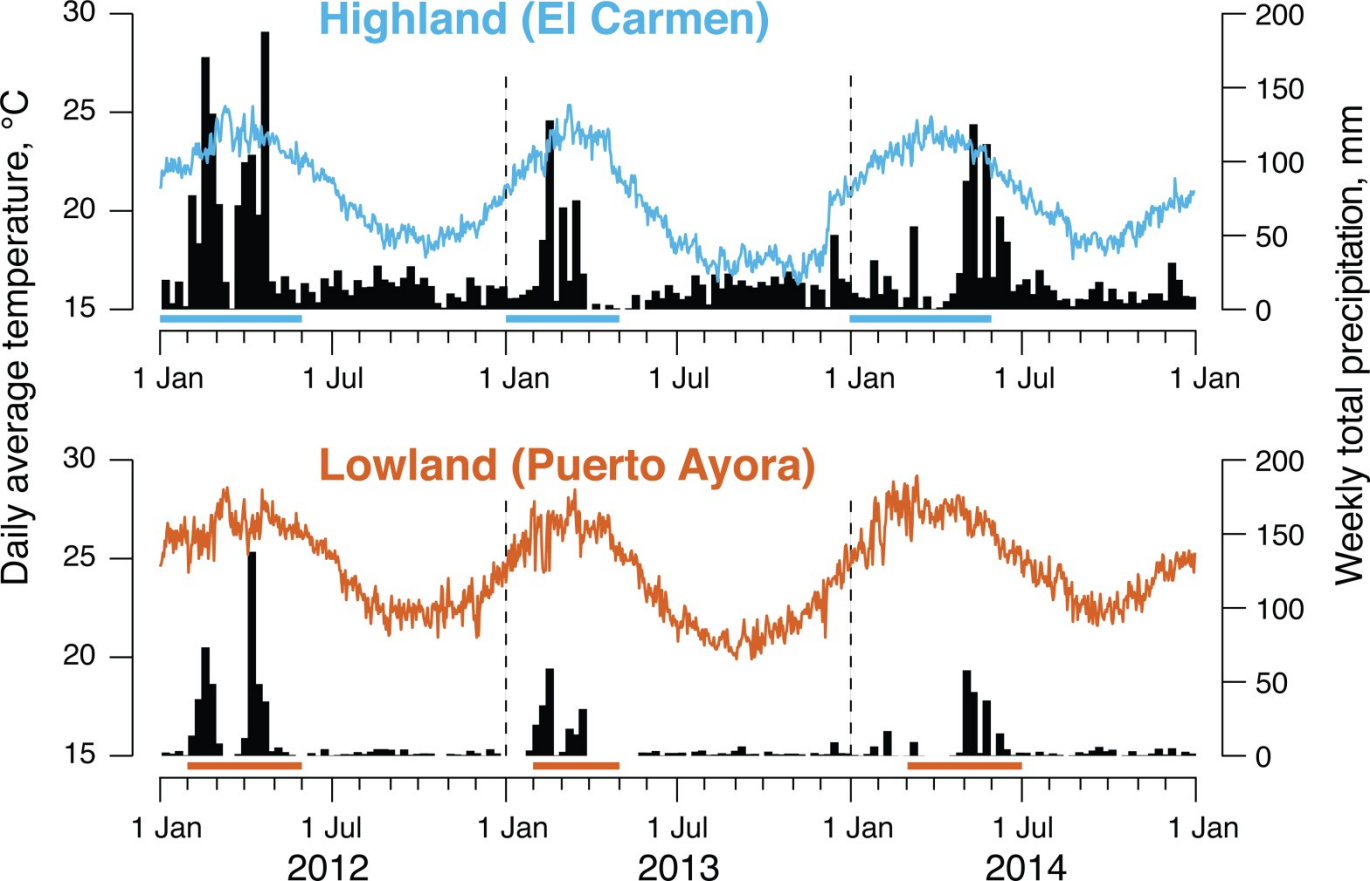
Daily average air temperatures and weekly precipitation totals recorded near the highland study site and the lowland study site on Santa Cruz Island, 2012–2014. Horizontal bars above calendar scales represent breeding seasons of known hosts (B. Fessl, pers. comm.).

Temperatures recorded in 2012–2014 at the lowland weather station were distinctly seasonal (Fig 1). Highest temperatures occurred in February–April, lowest in September–November, and the overall average was 24.5°C. Over the same period, average humidity level was 86.5%, but the humidity sensor appeared to be malfunctioning in 2013. Mean weekly precipitation was 6.3 mm, but occurred in distinct rainy seasons during February-April in 2012–2013, and May-June in 2014. The annual temperature pattern was similar at the highland station, except that the overall mean was 20.8°C, 3.7°C cooler than in the lowland zone (Fig 1). Average humidity level was 85%, and average weekly precipitation was 23.1 mm, 16.8 mm more than at the lowland station. Highland rainy seasons coincided with rainy seasons at the lowland site, but more rain fell during the June–December cool season at the highland site than at the lowland site (Fig 1).

### Abundance in fly traps

Traps were operated over 2½ years for 68 trapping intervals at the lowland site, and 66 intervals at the highland site. All combined, totals of 6,880 female and 2,189 male *P*. *downsi* were obtained during 27,848 trap-days, for overall catch rates of 0.25 females and 0.08 males per trap per day.

**Fig 2:**
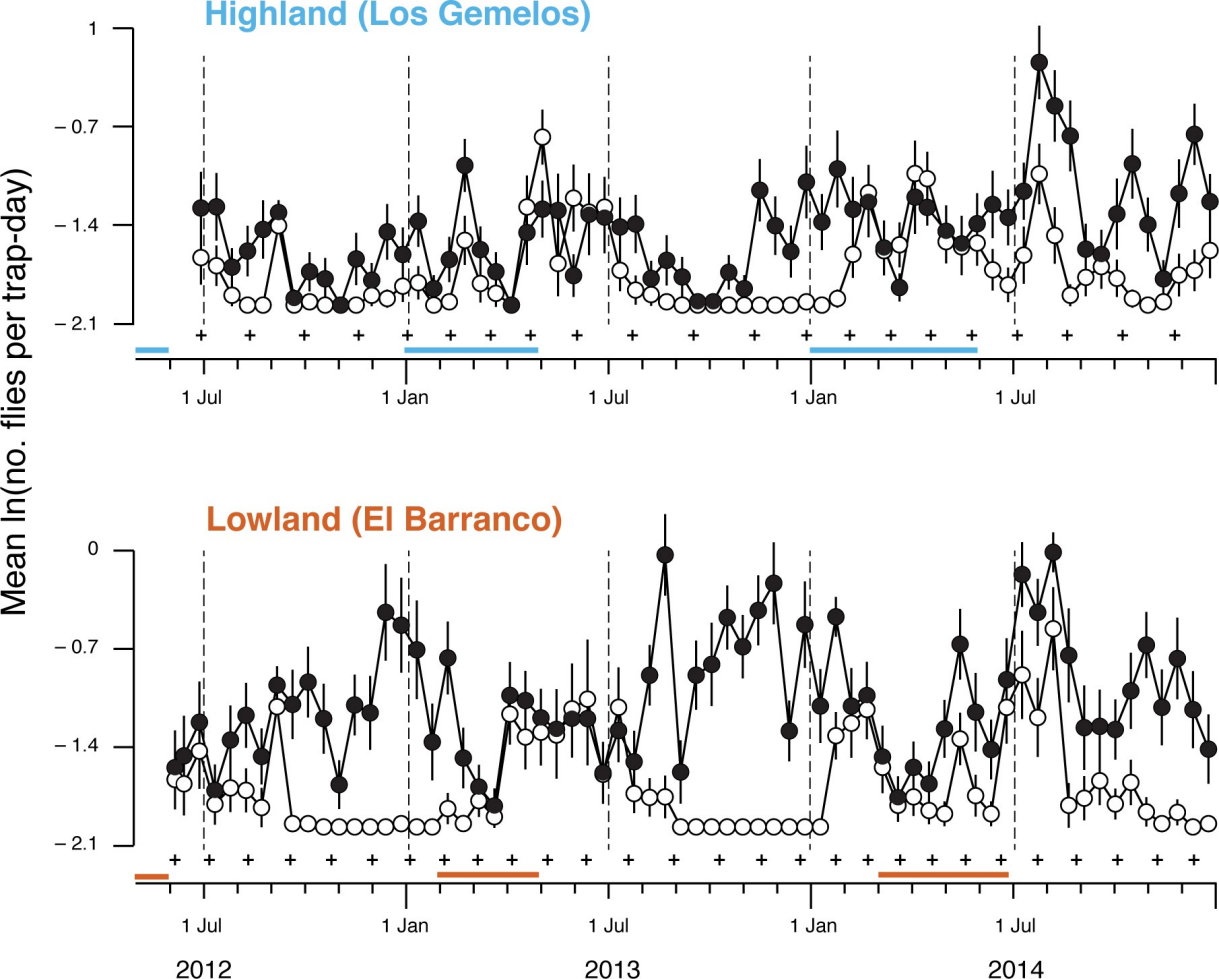
Catch rates of female (filled circles) and male (open circles) *P*. *downsi* at the highland and lowland sites, by date traps were emptied. Vertical bars around points are ± 2SEs of means (only visible when greater than symbol diameter), *n* = ~30 traps per point. Plus marks (+) delimit consecutive fly generations, estimated with a day-degree based generation time model. Horizontal bars above data scales represent breeding seasons of known hosts.

Catch rates indicated populations of adult *P*. *downsi* were continuously active at both sites for the duration of the study (Fig 2). Females were detected during all 68 trapping intervals at the lowland site, and 97% (64 of 66) of the intervals at the highland site. In contrast, males were detected during 73% of the intervals at the lowland site, and 76% of the intervals at the highland site. Absence of males coincided with the non-bird breeding seasons at both locations. Males were relatively more abundant in the non-bird breeding season of 2014, the year when rains came later, than in 2012 and 2013 (Figs 1 and 2). In contrast, females remained active—if not increased in numbers—after the two bird breeding seasons at both study sites (Fig 2).

Numbers of trapped flies were essentially random with respect to trap location: estimated variance components from study sites and trap locations within sites accounted for less than 10% of overall variation in catch rates (Table 1). Moreover, ranked abundance among locations was inconsistent from one trapping interval to the next. Temporal autocorrelation among trap locations was weak overall, never exceeded 0.2, and diminished with increasing 2-week lags. Thus, we treated individual trap locations as random effects in subsequent analyses.

**Table 1:** Variation in catch rates, partitioned among study sites, trap locations within sites, and residual error from variation among trapping dates across locations.

Source	St. Dev	Variance	% of total
Sites (*n =* 2)	0.144	0.021	3.4
Locations in sites (*n =* 30)	0.191	0.036	5.8
Residual error	0.751	0.565	90.8
	Total:	0.623	

The best fitting generalized additive mixed models (GAMMs) overall included both weeks (fitted as a Duchon spline with on average 10.40 estimated df) and weather. If only weeks or weather were included in the model then the ‘weeks’ model (weeks as a spline or as a fixed factor) was a better fit than the ‘weather’ model. The importance of ‘weeks’ and ‘weather’ suggests that broad scale seasonal, cyclic patterns and to a lesser extent, local climatic conditions both influence the catch rate. Furthermore, we found that these effects differ in lowland and highland sites and do not influence male and female *P*. *downsi* in the same way, as GAMMs, which included interactions between weather/week and sex and site, were consistently better fitting than those without (Table 2).

**Table 2:** Analysis of variance and estimated regression coefficients taken from generalized additive mixed models for average daily catch rates of *P*. *downsi*, with fixed effects of fly sex and site and comparing models where time (study week) is treated as a fixed factor or fitted using a smoothing spline (edf = estimated degrees of freedom for the spline). Also compared are models where interactions between weeks and site/sex and weather and site/sex are fitted (* p < 0.05, ** p < 0.01, *** p < 0.001).

Model	df	AIC	Term	edf	Coef ± SE	t	F
Week	2, 7927	753.59	Intercept		0.062 ± 0.008	7.99***	
(fixed)			Week		0.001 ± 0.000	11.05***	122.10***
Week	2, 7927	849.92	Intercept		0.118 ± 0.007	17.05***	
(ds spline)			Week	10.71	-0.002 ± 0.002	-0.85	29.61***
Weather	4, 7925	928.16	Intercept		-0.053 ± 0.028	-1.93*	
			Rainfall mm		-0.001 ± 0.000	5.68***	23.33***
			Relative humidity %		0.001 ± 0.000	2.45***	32.28***
			Temperature°C		0.003 ± 0.001	4.83*	6.01*
Week and weather	5, 7924	746.41	Intercept	1	-0.132 ± 0.068	-1.94*	
			Week	10.72	0.001 ± 0.002	0.44	29.36***
			Rainfall mm		-0.001 ± 0.000	-5.05***	25.45***
			Relative humidity %		0.001 ± 0.000	4.99***	24.93***
			Temperature°C		0.007 ± 0.003	2.42*	5.85*
Week and weather by sex	10, 7919	-228.89	Intercept		-0.593 ± 0.076	-7.76***	
			Male | Week	10.25	-0.005 ± 0.002	2.84*	16.04***
			Female | Week	10.85	0.007 ± 0.003	-2.07*	46.16***
			Sex (Male)		0.025 ± 0.003	11.03***	126.96***
			Rainfall mm		-0.001 ± 0.0002	-3.03**	9.16**
			Sex | Rainfall mm		-0.0004 ± 0.0004	-1.08	35.56***
			Temperature°C		0.025 ± 0.003	7.58***	57.50***
			Sex | Temperature°C		-0.030 ± 0.003	-11.32***	128.11***
			Relative humidity %		0.003 ± 0.0003	8.99***	80.88***
			Sex | Relative humidity %		-0.003 ± 0.0004	-5.96***	35.57***
Week and weather by site	10, 7919	699.33	Intercept		0.221 ± 0.184	1.20	
			Site (Low)		-1.619 ± 0.343	-4.72***	22.31***
			High | Week	9.918	-0.002 ± 0.0031	-0.67	14.72***
			Low | Week	10.319	-0.0007 ± 0.002	-0.31	14.53***
			Rainfall mm		0.0007 ± 0.0003	-2.43*	6.16*
			Site | Rainfall mm		-0.003 ± 0.001	-4.47***	20.02***
			Temperature°C		-0.006 ± 0.009	-0.6	0.36
			Site | Temperature°C		0.0289 ± 0.0136	2.13*	4.55*
			Relative humidity %		-0.00007 ± 0.0003	-0.25	0.06
			Site | Relative humidity %		0.012 ± 0.002	7.08***	50.10***

Broad scale seasonal changes in the catch rate over weeks were different for males and females; fitting separate splines to ‘week’ for males and females drastically improved the model fit (Table 2; Fig 2). Daily catch rates of females and males also differed significantly across sites (Sex*Site: F_1, 7986_ = 359.12, p <0.0001). Weather also affected catch rates of males and females differently; only the effect of rainfall was consistent for males and females, as rainfall increased DCR decreased. As temperature and humidity increased the female catch rate increased, but for males the effect of humidity and temperature were negligible (Table 2). The trap rate fluctuated over weeks in an inconsistent way over the highland and lowland sites (Table 2), but site-dependent effects of climate were less ambiguous (Table 2). DCR was negatively related to rainfall across both sites, but the decline was steeper in the lowlands. Overall DCR increased with increasing relative humidity and temperature, but this only appeared to occur in the lowlands; in the highlands there was no discernable relationship between DCR and relative humidity or temperature.

### Reproductive status of females

**Fig 3:**
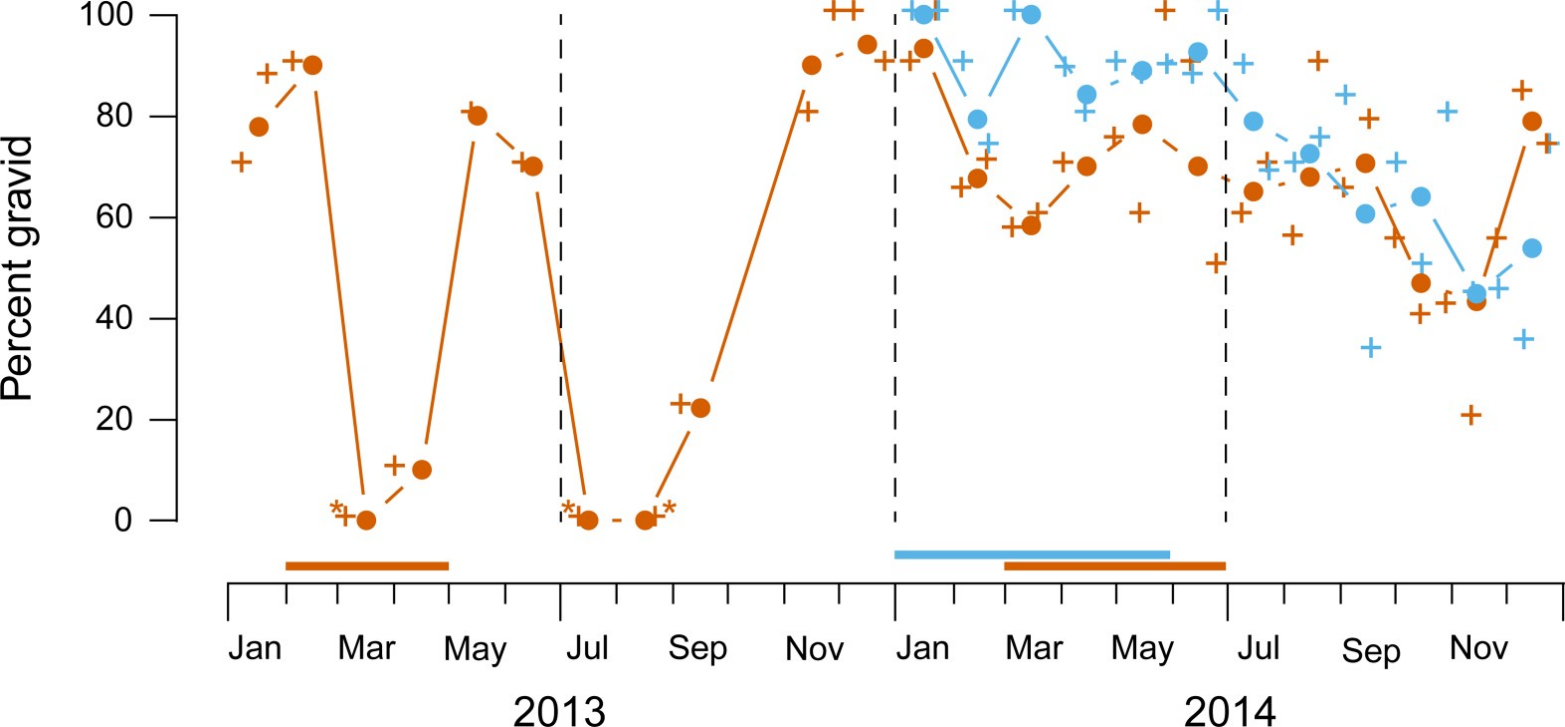
Frequencies of gravid (vs. undeveloped) *Philornis* females. Data from 2013–2014 at the lowland site are shown in orange, and data from 2014 at the highland site are shown in blue. Crosses are raw proportions of *n* = 7–19 females (or * *n* = 2). Filled circles with lines are pooled proportions plotted at midmonth, estimated with binomial regression. Horizontal bars above calendar scales represent breeding seasons of known hosts.

The majority (~71%) of 878 dissected females were gravid during both the bird breeding and nonbreeding seasons. Those females were from trap samples collected on 14 dates at the lowland site in 2013, and on 25 dates at each of the lowland and highland sites in 2014. Percent gravid females oscillated between zero and ~80% at the lowland site in 2013 (Fig 3). In 2014, levels at both sites decreased from ~90% to ~50% over the year. Monthly variation in proportions of gravid females was not consistent over site-years (binomial regression month in site-year: likelihood ratio *χ*^2^ = 83.53 w/34 df, *p* < 0.001), whereas monthly patterns in 2014 were the same at the lowland and highland sites (interaction between the two site-years and months: likelihood ratio *χ*^2^ = 14.28 w/11 df, *p* = 0.22). Timing of oscillations in 2013 and the comparatively smoother downward trends in 2014 seemed to be independent of timing of bird breeding (Fig 3).

Numbers of eggs per gravid female ranged from 1–47, the mean was 17.1, but counts were overdispersed (Poisson dispersion parameter = 7.33). The Poisson GLMM indicated that mean egg loads of non-mated and mated females were not different (likelihood ratio *χ*^2^ = 0.44 w/1 df, *p* = 0.50), and that means from the lowland and highland sites were not different (*χ*^2^ = 0.14 w/1 df, *p* = 0.70). Means among months within site years did vary significantly (*χ*^2^ = 38.73 w/18 df, *p* = 0.003), but the seasonal patterns in egg loads were weak, and not obviously related to rainfall or bird breeding seasons (not shown).

**Fig 4:**
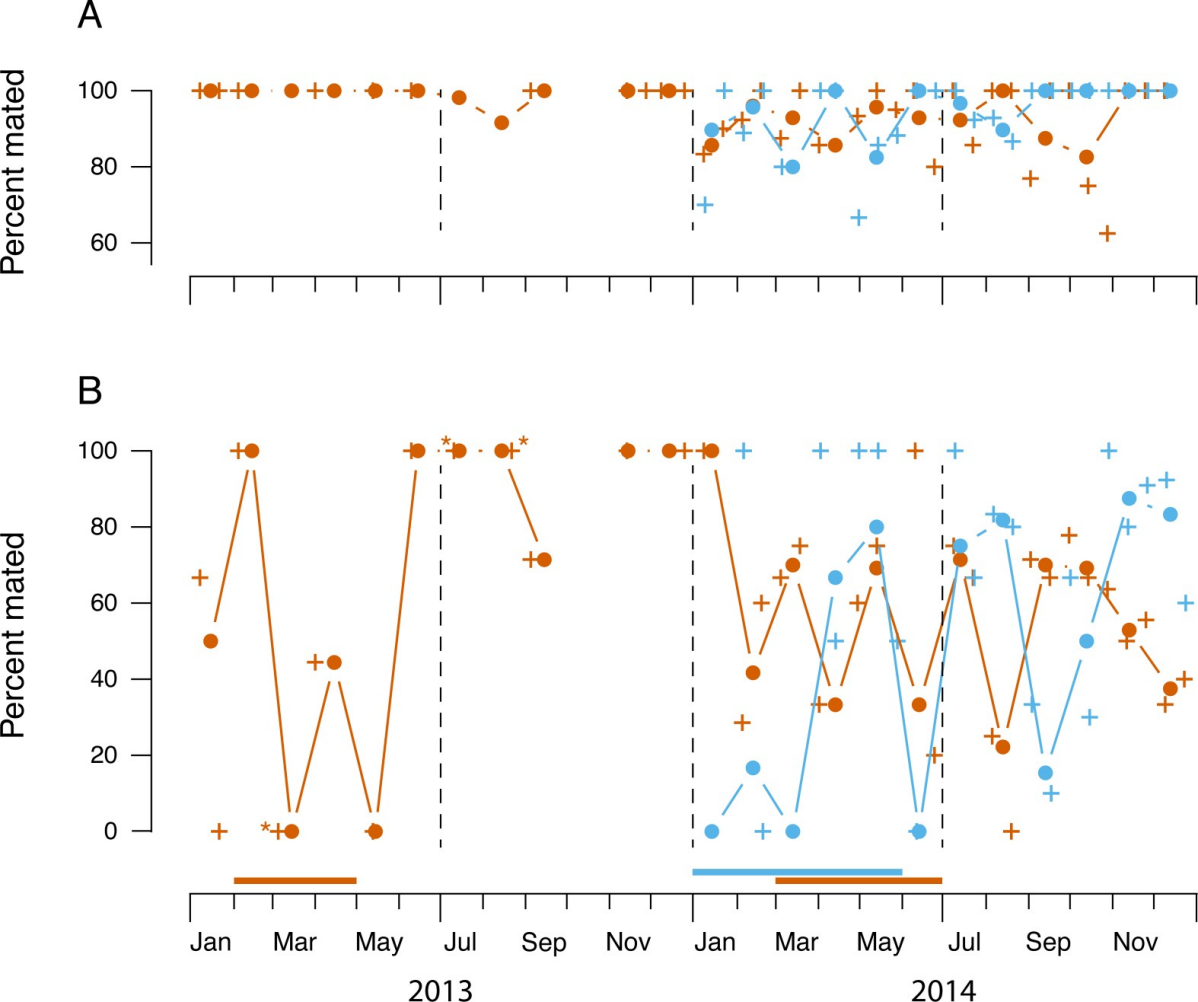
**Mating frequencies among gravid (A) and undeveloped (B) *Philornis* females.** Lowland (orange), highland (blue). Crosses are raw proportions of *n* = 7–19 females (or *n = 2). Filled circles with lines are pooled proportions plotted by midmonth, estimated with binomial regression. Horizontal bars above calendar scale indicate breeding seasons of known hosts.

Spermathecal squashes indicated that mating frequencies were consistently high among the dissected females (Fig 4); 94% of 620 gravid females from all three site-years had been mated and 60% of the 258 undeveloped females were mated (effect of reproductive stage:*χ*^2^ = 40.81 w/1 df, *p* < 0.0001). Mating frequencies among gravid females (Fig 4, top) were consistently above 80% in all three site-years, and statistically independent of bird-breeding seasons, locations and site-years. Interestingly, mating frequencies were independent of concurrent catch rates of females (*χ*^2^ = 2.53 w/1 df, *p* = 0.11). Mating frequencies among undeveloped females at the lowland site in 2013 (Fig 4, bottom left), varied erratically from month to month, but samples were infrequent and sample sizes too small to discern clear trends. Patterns at the two sites in 2014 were also erratic, but appeared to be independent of bird breeding activity.

### Sex ratio of flies emerging from nests

A total of 239 nests yielded 7,998 larvae, pupae or empty puparia, and 3,321 of the larvae and pupae were reared to adulthood and sexed. A binomial GLMM with an OLRE indicated proportion male did not differ between the two host species (*χ*^2^ = 0.77 w/1 df, *p* = 0.38), among the 19 weeks (*χ*^2^ = 8.65 w/18 df, *p* = 0.97), and among the three years (*χ*^2^ = 2.51 w/2 df, *p* = 0.28). Furthermore, proportion male was independent of the density of immature *P*. *downsi* (*χ*^2^ = 1.55 w/1 df, *p* = 0.21), and proportion sexed (*χ*^2^ = 0.48 w/1 df, *p* = 0.49). We did find that the proportion of male *P*. *downsi* decreased as the number of *P*. *downsi* per chick in the nest increased (*χ*^2^ = 5.82 w/1 df, *p* = 0.02). Overall, when every sexed specimen was given an equal weight, 95% confidence intervals for the proportion male was 0.45 ± 0.02, a slight bias toward females.

### Levels of nest parasitism and matching catch rates

The average Warbler Finch nest (± SE) at the highland site in 2014 contained 24.4 ± 1.2 (*n =* 93) immatures of *P*. *downsi*. The average Small Tree-finch nest contained 30.9 ± 2.7 (*n =* 70). Daily catch rates of female *P*. *downsi* in traps interspersed among the nests averaged 0.11 females per trap-day. A Poisson GLMM indicated counts of immatures per nest varied significantly between the two bird species (*χ*^2^ = 24.78 with 1 df, *p* < 0.001), and increased with time from egg hatch to failure or fledging (*χ*^2^ = 45.41 with 1 df, *p* < 0.001). However, counts of immatures were independent of week of egg hatch (*χ*^2^ = 16.61 with 13 df, *p* = 0.21), number of chicks in the nest (*χ*^2^ = 3.73 with 1 df, *p* = 0.053) and matching log_2_ DCRs of females in traps (*χ*^2^ = 3.57 with 1 df, *p* = 0.06). The same conclusions were reached when dates for trapping intervals were advanced or retarded by one or two weeks relative to the known nest hatching dates.

### Population dynamics of *P*. *downsi*

The development time model predicted the shortest egg-to-egg generation time would have been 29 days if computed from daily average temperatures at the lowland weather station, starting in February (S1 Appendix). In contrast, the longest generation time would have been 56 days using temperatures at the highland station, starting in September. Sequential calculations from first to last trapping dates indicated that 27 complete generations and a partial 28^th^ elapsed at the warmer lowland site, whereas 20 and a partial 21^st^ were likely at the cooler highland site (Fig 2).

**Fig 5:**
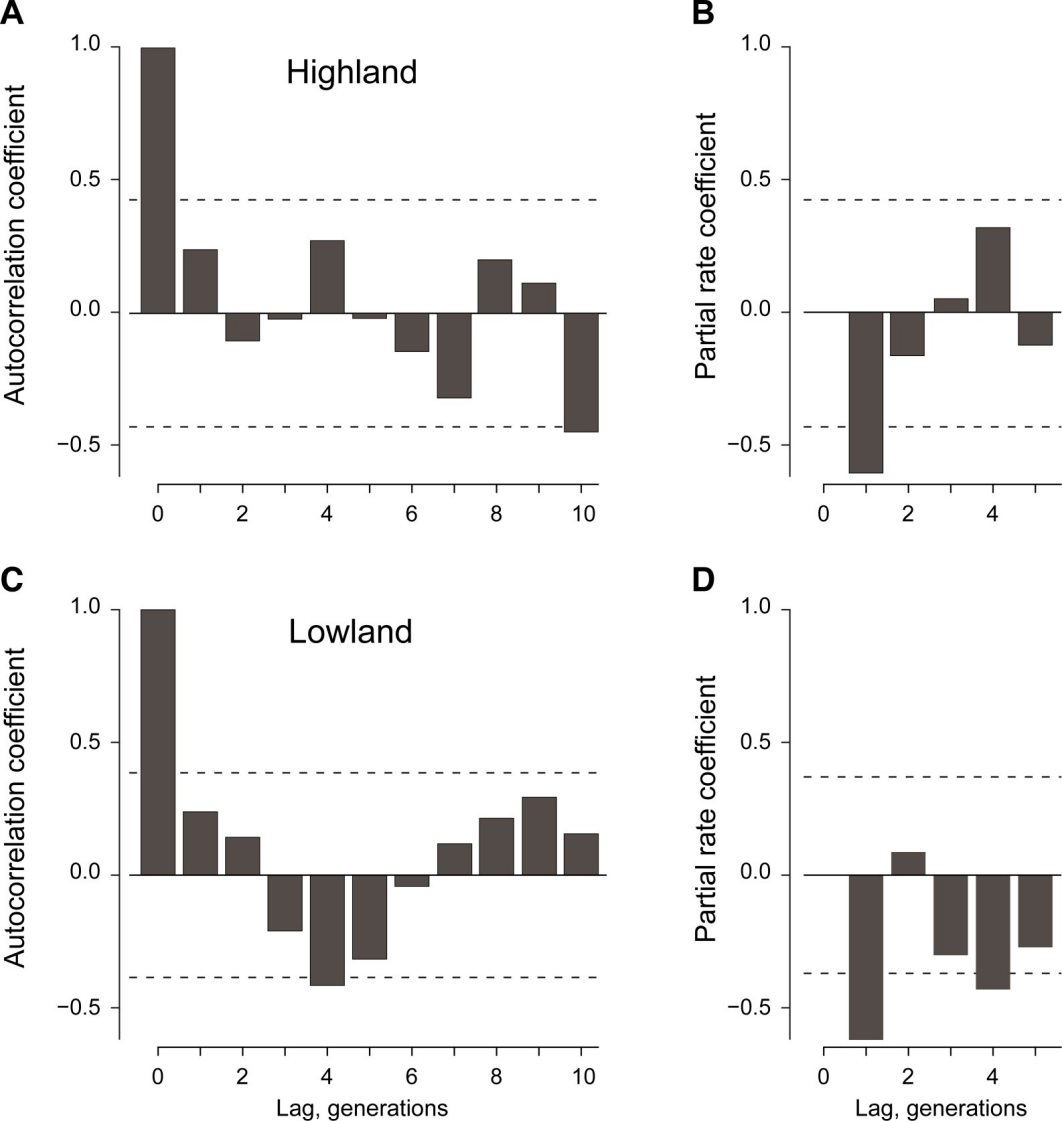
**Temporal autocorrelation coefficients (A, C) and partial rate correlation coefficients (B, D) among consecutive generations of female *P*. *downs*i at the highland and lowland study sites.** Coefficients outside the dashed horizontal bands in each panel are significantly different from zero (*α* = 0.05).

Time series analysis of population growth rates, defined as changes in mean log_2_ catch rates during consecutive generations, revealed that ACFs at the lowland site and the highland site damped rapidly, and oscillated around zero (Fig 5A and 5C, respectively). The corresponding PRCFs from the two sites (Fig 5B and 5D) indicated the coefficients with lag 1 were significantly negative, whereas later ones out to 5 generations were not, with exception of the significantly negative value for the 4-generation lag at the lowland site (Fig 5D). Values for PRCFs beyond lags of 5 generations (not shown) were deemed unreliable, because of small sample sizes. The ACF and PRCF patterns were consistent with the hypothesis that simple density dependent regulation is the chief factor regulating populations at both sites.

**Fig 6:**
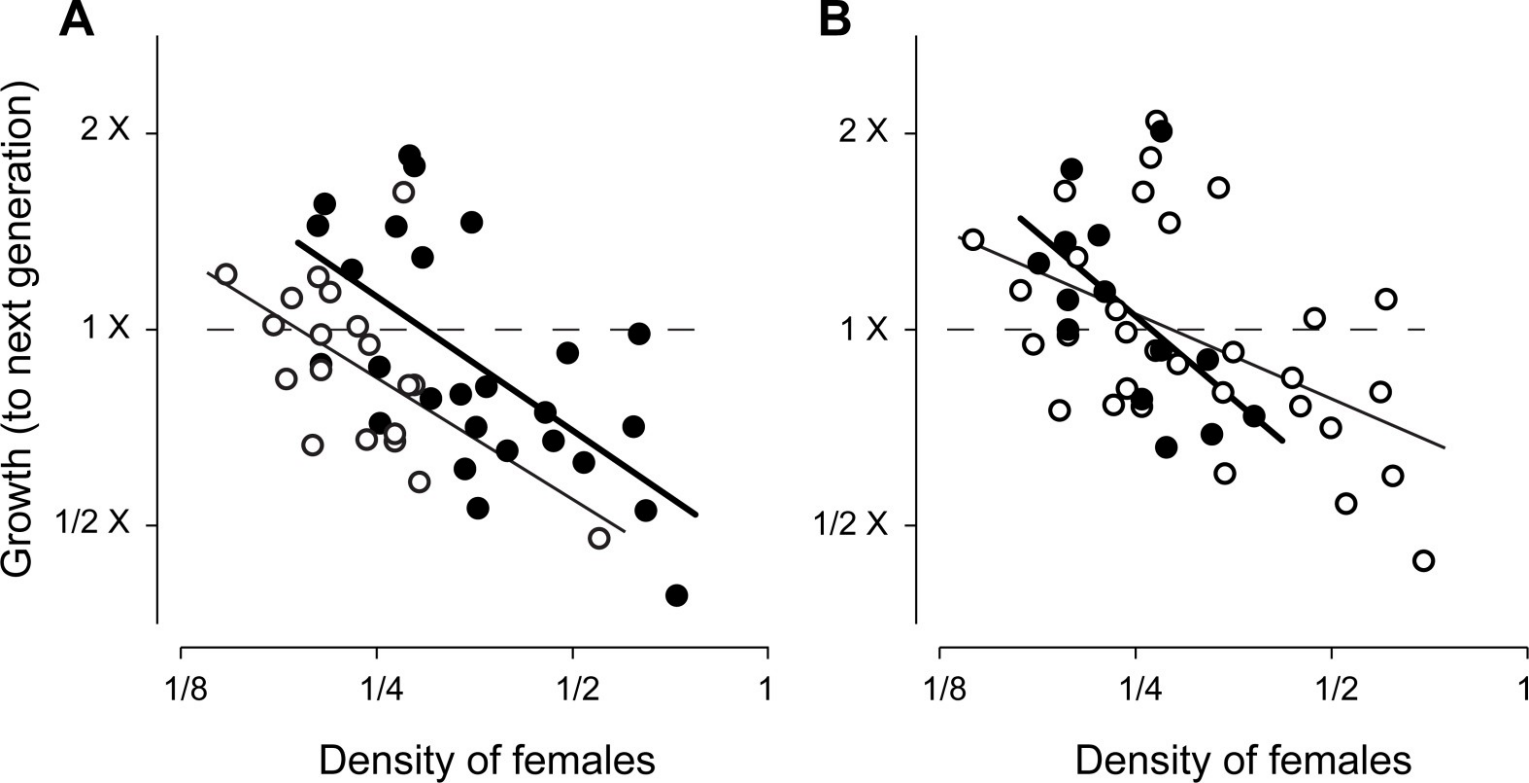
Inverse relations between growth rates and densities of female *P*. *downsi*, expressed as Log_2_-transformed daily catch rates. A: Growth at lowland site (filled circles) versus highland site (open circles), seasons combined; B: growth during bird breeding season (filled circles) versus non-breeding season (open circles), locations combined. Solid lines are least-squares regression lines fit to data in each subgroup separately. Horizontal dashed lines indicate population replacement, and intersections with regression lines indicate estimated carrying capacities, which were greater at the lowland site, but independent of bird breeding season.

Finally, regression analysis indicated population growth rates were inversely related to density of parental fly generations at both the lowland and highland sites (Fig 6A; Table 3). In contrast, growth rates were independent of breeding season and of all of the three meteorological variables (Fig 6B; Table 3). Fitted regression coefficients (Table 3) were used to estimate equilibrium densities at the two study sites, based on generational limits from the 11-d generation time models. Mean equilibrium catch rates ± approximate 95% confidence limits, back-transformed to arithmetic scale, were 0.39 ± 0.22 females per trap-day at the lowland site, and 0.21 ± 0.17 females per trap-day at the highland site.

**Table 3:** Analysis (ANOVA) of population growth of *P*. *downsi* females in relation to effects of biotic and abiotic factors.

Source	SumSq	Df	F	Pr(>F)	Term	Coefficient^a^	SE
					Intercept	1.506	1.699
**Maternal density**	**4.20**	**1**	**19.08**	**<0.001**	**Density**	**-0.719**	**0.165**
**Study site**	**1.21**	**1**	**5.48**	**0.025**	**H(vs. L)**	**-0.742**	**0.317**
Bird breeding season	0.19	1	0.85	0.362		0.234	0.923
Avg Temp,°C	0.36	1	1.61	0.212		-0.074	0.058
Avg % RH	< 0.01	1	0.01	0.902		-0.003	0.010
Avg rain, mm	0.01	1	0.054	0.823		-0.009	0.043
Residuals	8.37	38					

^a^ Change in mean log_2_ daily catch rate (DCR) between consecutive generations, per unit change in continuous source variables, or as difference between highland (H) vs. lowland (L) sites, or bird non-breeding *vs* breeding season. *R*^2^ = 0.31.

## Discussion

### Seasonal abundance in lowland and highland habitats

Our findings demonstrate for the first time that gravid, inseminated female *P*. *downsi* are active during and between the bird-breeding seasons. Female *P*. *downsi* were trapped at both the dry lowland site and the humid highland site on Santa Cruz Island throughout 2012–2014. This included two hot seasons (~January–May), when most bird hosts [25] nest, and three cool seasons (~June-December), when host nesting activity was typically rare. Persistence of females at the lowland site during the cool and drier season demonstrates *P*. *downsi* can tolerate arid, dry habitats even when hosts are scarce. This was especially notable in 2014 when there was 13 months between substantial precipitation events and bird breeding activity started later than usual. Rainfall promotes vegetation growth, which provides passerines with the resources needed for breeding [25].

At both sites, trap catch rates of male *P*. *downsi* were more seasonal than catch rates of females. Males increased after breeding seasons began in each site-year, with periods of decrease and apparent absence occurring between breeding seasons, in particular in 2012 and 2013. Seasonality of males may be explained by three non-exclusive hypotheses. First, scarcity of males may have been an artifact of males having a higher flight threshold temperature than females. It is notable that male catch rates were essentially zero at the cooler highland site from August to November when mean daily temperatures were below ~19–21°C. However, catch rates at the warmer lowland site were only zero when mean air temperatures were 4°C warmer, below ~22–26°C. Dissimilarity between catch rates and temperature at the two sites, and the lack of any effect of temperature on male trap rate, is evidence against this flight threshold hypothesis.

A second hypothesis is that males become less attracted to papaya bait between bird breeding seasons. Although field studies in Galapagos have confirmed that young and old males are attracted to yeasts and fermenting fruits [18,26], it is possible that males outside the bird breeding season need less food or seek a different food than females, as occurs with some muscid, calliphorid and tephritid fruit flies [27,28,29].

A third hypothesis is that abundance of males and females may truly differ seasonally at both sites. One mechanism could involve differential recruitment of emerging adult males and females. While we found that approximately 56% of flies reared from nests of two bird species at the highland site were females, it remains to be seen whether sex ratios are the same in the lowland habitats, and whether they are different during intervening cool seasons at both sites. A second mechanism could involve differential longevity of females in the interbreeding seasons, and/or a possible cool, dry season aestivation or diapause. Female *P*. *downsi* have been known to live for up to 265 days and males up to 188 days in the laboratory on fruit-protein diets (MPL). A slight upward trend in mated females with undeveloped eggs between bird breeding seasons could suggest developmental arrest, egg resorption or dumping as observed in other species of flies [30,31,32]. If males are unable to live as long as females, or if they do not have a similar developmental arrest, then this mechanism could lead to decline in relative frequencies of males through mortality at the end of the bird breeding season. A third mechanism could involve differential dispersion of males and females on the landscape, a subject that could not be addressed with the trap layouts used in this study.

It is notable that catch rates and equilibrium densities of female *P*. *downsi* were generally greater at the dry lowland site than at the humid highland site. These results differ from the trends observed in nests surveyed on Santa Cruz Island in previous years that either did not find a marked difference in prevalence or density of immature *P*. *downsi* found in nests in the highland and lowland regions [33,34] or found higher densities of parasites in nests in the humid highlands [35]. This evidence, along with our findings that there was no correlation between trap catch rates and *P*. *downsi* density in nests near traps, suggests that different processes are driving the catch rates of adult *P*. *downsi* in traps and the abundance of immatures in nests.

One hypothesis for catch rates of adult females being greater at the lowland site may be that the difference is an artifact of differential attractiveness of papaya-baited traps in the two landscapes. Adult *P*. *downsi* feed on fermenting fruits of a range of native and introduced plant species (P. Lahuatte, pers. comm.). Trap catch rates may be influenced by differences in the diversity, abundance and quality of fruiting plants found at the lowland and highland sites [36]. It is notable that the understory at the highland site is dominated by the invasive blackberry *R*. *niveus*, which fruits year-long [16,36,37], and is a known food of *P*. *downsi* (P. Lahuatte, pers. comm.). Fermenting blackberry is also highly attractive to *P*. *downsi* (CEC). More needs to be learned about the efficiency of baited McPhail traps and the effects of neighboring vegetation on trap efficiency before catch rates can be equated confidently with absolute densities of adults in different habitats.

In the future, thorough nest censuses to quantify absolute nest abundance, and simultaneous sampling to quantify parasite intensity could be used to test the hypothesis that recruitment of *P*. *downsi* is greater in lowland habitats as well as to test whether these differences can be found on islands with and without anthropogenic influences. Simultaneously, longevity of female and male flies in each habitat could be compared using age-grading or mark-release-recapture methods to assess habitat suitability for adult flies and efficiency of McPhail traps or other devices used to measure abundance.

### Weather and flight activity of *P*. *downsi*

We hypothesized that catch rates of female and male *P*. *downsi* in McPhail traps would be related partially to concurrent measures of temperature, humidity and precipitation at each location. Flight activity of insects is well known to be affected by meteorological conditions, particularly ambient temperature [38,39,40], and weather varies seasonally in the lowlands and highlands of Santa Cruz Island. Our regression analyses indicated that average daily catch rates of both males and females decreased modestly with increasing rainfall (around 1.3 mm per day). Catch rates of females increased with increasing average daily temperature (around 22.3°C) and relative humidity (around 85%), whereas the effect on male catch rates was negligible. Variation in weather accounted for 40% of the overall variation in catch rates of flies over the 2½ years of trapping at the two study sites. Sources of the remaining variation are not well understood, but are likely to involve mechanisms that confer first-order density dependence.

### Density dependence and weather effects

Three lines of evidence from our study indicate that *P*. *downsi* populations at both study sites consist of continuous, multivoltine, overlapping generations. First, females were detected in virtually all 2-week trapping intervals spanning the full 2½ years of study. Second, dissected female flies from 2013–2014 were a mixture of gonotrophically undeveloped and gravid individuals, and mating frequencies were high and did not differ markedly over the year. Third, assuming our estimates of generation times are approximately correct, analysis of growth rates with ACF and PRCF functions revealed stability and first-order dynamics; periods of increase and decrease of flies were not in synchrony with numbers of generations at either site.

Our results suggest that *P*. *downsi* is capable of producing 4–5 overlapping generations during the bird breeding season at the warmer, drier lowland site and 3–4 generations at the cooler, humid highland site, but that generational means are not strongly influenced by temperature, humidity and rainfall. The observed persistence and positive growth rates of *P*. *downsi* outside the bird breeding seasons at both sites indicate *P*. *downsi* populations could be, at least in part, supplemented in “non-breeding” seasons by isolated bird nesting activity. This is supported by observations of occasional nests at the highland site during the cool seasons of 2012–2014. These nests were infested with *P*. *downsi*. Furthermore, longer rainy seasons can prolong bird-nesting activities such as that observed in 2014 at both highland and lowland sites. Regardless, the extremely low numbers of males in our traps during the non-bird breeding season compared to the high frequencies of mated gravid females suggests that females are persisting in the absence of males and hosts.

Time-series analyses provided evidence that populations of female *P*. *downsi* at both study sites were regulated and stable, and that the regulatory mechanism was mostly governed by simple direct density dependence. Reproduction appears to be limited by host availability, which is markedly seasonal in Galapagos. Host availability and host distribution, in turn, may influence how many *P*. *downsi* females opt to lay their eggs in a single nest and how many eggs are deposited by each female, leading to regulatory effects through larval competition. An earlier study using molecular techniques [41] found that nests on Santa Cruz Island contained progeny of multiple flies and that the average contribution of eggs by a single female was low (five eggs) even though females had the capacity to lay eggs in higher numbers in a single laying event (up to 24 eggs). This low genetic relatedness suggested that flies had either mated with multiple males and/or that the larvae were from different females, either of which would be expected to promote competition for resources.

Obvious mechanisms that could give rise to direct density dependence are that crowding of larvae on chicks could reduce larval survival. High larval numbers in turn causes early chick death and reduces availability of food for larvae [14]. Our findings with regard to the sex ratio of flies in nests suggest that larval competition may be an important factor. The sex ratio became more female biased as the number of larvae per chick increased. This could represent sex differences in survival under increased competition and/or facultative sex allocation under a Trivers-Willard type process [42]. Regardless of the mechanism, it suggests that increased larval competition due to crowding represents an important pressure regulating the population dynamics and ecology of *P*. *downsi*.

Larval crowding could occur because of an increase in absolute numbers of ovipositing *P*. *downsi* in an area, a decrease in absolute number of nests and chicks in the same area, or both. Crowding would be expected to occur at the beginnings and ends of bird breeding seasons, when nest and chick abundance would naturally be relatively low. Thus far, such a pattern in *P*. *downsi* intensity has not been observed over bird breeding seasons in other studies on Santa Cruz [19], but the absence of such a trend may be because nests at the tails of breeding seasons have not been monitored sufficiently. Alternatively, high *P*. *downsi* intensity in nests might be expected in nests in a dry year (with low host density) if it has been preceded by a wet year with higher host density and high fly numbers. This was not found in a small study in the dry zone of Santa Cruz [43]. Additional molecular screening of larvae in nests, in particular during periods of high and low nest densities under different climatic conditions, would provide further insights into the dynamics of this nest parasite.

Time-series analyses suggest that more subtle interspecific interactions may influence population dynamics, as ACF and PRCF coefficients for the 4-generation lag were statistically significant in some cases. The source of any interspecific interactions that might produce such a pattern warrants further attention. It is unlikely that larval and pupal parasitism by hymenopterans explains this, as parasitism rates are low and do not influence *P*. *downsi* density in nests [5]. Moreover, we know of no competitive interactions with other nest parasites in Galapagos, but comparisons with *P*. *downsi* in its native range, where competitors and parasites likely contribute to population dynamics [5,10] would be a valuable future endeavor.

### Conclusions and implications for landbird conservation

Our study suggests that *P*. *downsi* populations are density-dependent. Thus, according to theory [22–24], *P*. *downsi* populations are very likely dependent on host supply, which in this system is the number of bird nests and nestlings. Additional studies will enable us to confirm this. An interesting question is how this theory translates to bird communities that are impacted by a highly successful invasive parasitic fly such as *P*. *downsi* that is able to persist even when host numbers decline and, which is laying its eggs earlier in the nesting process (and laying progressively higher numbers of eggs in nests), causing nestlings to die earlier and at higher rates [13,14]. At our study sites gravid, inseminated females were abundant throughout the ~ seven-month cool period between bird breeding seasons suggesting that large numbers of flies continue to search for hosts even when conditions are less favorable. Importantly, this may have serious implications for the success of any nests that are produced during the cool season or at the very beginning and end of the bird breeding season. Higher competition for nests at these times could mean greater fly density in nests and greater impact.

Species that are most vulnerable to *P*. *downsi* parasitism such as smaller bodied arboreal finches (*Camarhynchus* spp.) are particularly at risk because the number of *P*. *downsi* larvae per nest can be very high and brood size is small [5]. Field studies and population viability analyses suggest that *P*. *downsi* has already played a major role in the decline of the two Critically Endangered island endemics, Medium Tree-finch and Mangrove Finch [44,45,46,47]. Even for more common species like the Medium Ground-finch, there are predictions of extinction within the next 100 years [48]. In contrast, larger-bodied birds such as the abundant Galapagos Mockingbird on Santa Cruz, the endangered Floreana Mockingbird, *M*. *trifasciatus*, and the Vegetarian Finch appear to be more resilient to parasitism by *P*. *downsi* than most smaller-bodied finches [49,50,51] (except in particularly dry years [52]). This has implications for their role as reservoir hosts. Density-dependent models predict that infestation rates should decline as bird density declines, but only if the population is not sustained by a less vulnerable reservoir host [53,54,55].

From a management perspective, it may be beneficial to target early-nesting reservoir hosts that are found in the vicinity of the nests of endangered bird species. Nests of the most endangered bird species are currently being protected by injecting permethrin into the base of the nest where larvae reside [5]. Extending this program to treat nests of reservoir hosts could be advantageous. However, this technique is labor intensive and does not rule out the possibility that flies could migrate from surrounding areas; little is still known about the movement patterns of *P*. *downsi* [5]. The development of an effective and long-lasting lure for trapping is underway [26,56,57], and other than helping manage *P*. *downsi* in high priority areas, will also provide a more effective tool for understanding the population ecology of *P*. *downsi* in the different vegetation zones found in Galapagos and on human inhabited and uninhabited islands. This information is crucial for wider reaching control programs such as biological control or Sterile Insect Technique that are being considered to reduce the impacts of this invasive parasite before it drives any of its hosts extinct [5,9].

## Supporting information

S1 Appendix Models for calculating generation time of *Philornis downsi* on Santa Cruz Island. (DOCX)
