# Supplementary material for: Population dynamics of an invasive bird parasite, Philornis downsi (Diptera: Muscidae), in the Galapagos Islands
Source: PLoS One. 2019 Oct 18;14(10):e0224125. doi: 10.1371/journal.pone.0224125 (PMC6874344; doi:10.1371/journal.pone.0224125)
Supplement: S1 Appendix — (DOCX) [file pone.0224125.s001.docx]

**S1 Appendix. Models for calculating generation time of *Philornis downsi* on Santa Cruz Island**

As a first approximation, we defined generation time of *P. downsi* as the time it takes a median female to develop from egg to pupa, plus time to develop from pupa to adult, plus time needed to produce a first batch of eggs (Lincango and Causton, 2008; Kleindorfer *et al*., 2014; Lahuatte *et al.*, 2016; M. Bulgarella pers.comm. 2016).

Time for development from egg to pupation (L) was set at a constant number of days, regardless of ambient temperature, because chick body heat maintains nest temperature at a level that is approximately constant (Sage *et al*., 2018). To bracket for variation in timing of fly oviposition, chick hatch, fly pupation and fledging, we tried values for *L* that ranged from 8 days to 15 days. However, preliminary analyses indicated that conclusions about population dynamics were insensitive to the 7-day difference, so we set *L* at 11 days, in the middle of the range.

In contrast, pupal development times (*P*) should vary inversely with ambient temperature, because pupae remain in unheated nests after fledging. Time for pupal development was set at 202 degree days above an arbitrary lower developmental threshold of 5 °C. These values were based on pupae reared at three different temperatures (Lincango and Causton, 2008; Kleindorfer *et al*., 2014; M. Bulgarella pers. comm. 2016).

Similarly, time for free living females to develop a batch of eggs (*E*) should also vary inversely with ambient temperature, as occurs with other muscid flies (Elvin and Krafsur 1984; Lysyk, 1991, 1998). Duration of *E* was set at 127 degree days above 5 °C, based on one unpublished observation (CEC).

Daily degree day increments (*D_i_*) toward *P* and *E* were calculated from mean daily temperature records (*T_i_*) at a chosen weather station, as *D_i_* = *T_i_* - *k* for *T_i_* > *k*, or 0 if otherwise.

Daily *D*s were then summed over consecutive days, and days required for development of pupae (*I_P_*) and eggs (*I_E_*) after an arbitrary starting date were then obtained by counting the number of days needed to accumulate their respective total degree days, *P* and *E*.

Finally, duration of a generation after an arbitrary starting date was then calculated as *G* = *L* + *I_P_* + *I_E._*

To visualize predictions of the model for temperatures extant on Santa Cruz Island, we used three-year records of daily mean temperatures from the lowland and highland stations to calculate generation times from arbitrary starting dates spanning the duration of the study. Short gaps of missing temperatures were filled by averaging observations from adjacent days. Results are shown in S1 Fig.


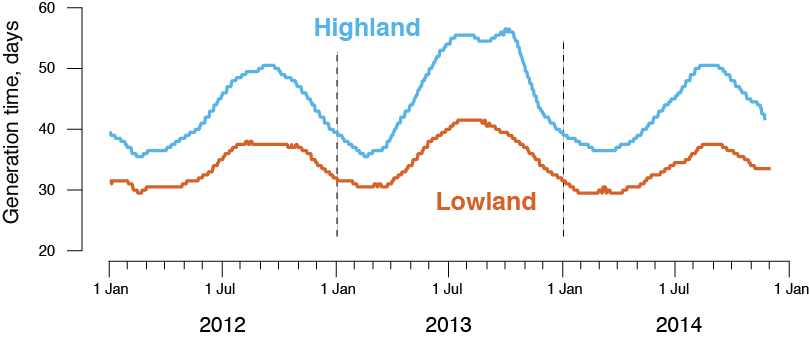


**S1 Fig.** Generation times (egg-to-egg) in days from each day forward, predicted with a temperature driven development time model and temperature records near the highland and lowland study sites (see text).

Finally, we used the model to estimate dates for beginnings of consecutive generations, starting on the first trapping date at each study site, which thereby divided the calendar into generational intervals delimited by plus signs (+) along the calendar scales of (see Fig 2).

**References**

1. Lincango P, Causton C. Crianza en cautiverio de *Philornis downsi* en las Islas Galápagos. Technical report. 2018. Charles Darwin Foundation, Puerto Ayora, Galapagos, Ecuador.

2. Kleindorfer S, Peters KJ, Custance G, Dudaniec RY, O'Connor JA. Changes in *Philornis* infestation behavior threaten Darwin's finch survival. Current Zoology. 2014; 60: 542-550.

3. Lahuatte PF, Lincango MP, Heimpel GE, Causton CE. Rearing larvae of the avian nest parasite, *Philornis downsi* (Diptera: Muscidae), on chicken blood-based diets. Journal of Insect Science. 2016. 16: 84: 1-7. <https://doi.org/10.1093/jisesa/iew064>

4. Sage R, Boulton RA, Lahuatte PF, Causton CE, Cloutier R, Heimpel GE. Environmentally cued hatching in the bird‐parasitic nest fly P hilornis downsi. Entomologia Experimentalis et Applicata. 2018; 166:752-60.

5. Elvin MK, Krafsur ES. Relationship between temperature and rate of ovarian development in the house fly, *Musca domestica* L.(Diptera: Muscidae). Annals of the Entomological Society of America. 1984; 77:50-5.

6. Lysyk TJ. Use of life history parameters to improve a rearing method for horn fly, *Haematobia irritans irritans* (L.)(Diptera: Muscidae), on bovine hosts. The Canadian Entomologist. 1991; 123:1199-207.

7. Lysyk TJ. Relationships between temperature and life-history parameters of *Stomoxys calcitrans* (Diptera: Muscidae). Journal of Medical Entomology. 1998; 35:107-19.
